# IRAK-M Knockout Exacerbates Inflammation in Mice Infected with *Streptococcus equi* subsp. *zooepidemicus*: Observation in the Jejunum

**DOI:** 10.3390/microorganisms14092068

**Published:** 2026-09-16

**Authors:** Yuxin Che, Yi Xie, Xinyi Qiu, Junxi Lin, Yixin Xiu, Taoyu Yu, Xiaoshu Zhan, Jiedan Liao, Yunfei Huang, Yaqiong Ye, Ziteng Deng

**Affiliations:** 1 School of Animal Science and Technology, Foshan University, Foshan 528225, China; 2 Foshan University Veterinary Teaching Hospital, Foshan University, Foshan 528225, China

**Keywords:** *Streptococcus equi* subsp. *zooepidemicus*, jejunum, interleukin-1 receptor-associated kinase M, NF-κB, macrophage

## Abstract

*Streptococcus equi* subsp. *zooepidemicus* (SEZ) causes severe infections, but the role of the negative regulator interleukin-1 receptor-associated kinase M (IRAK-M) in SEZ-induced inflammation is unclear. In this study, male C57BL/6 mice received a single intraperitoneal injection of SEZ at 2 × 10^7^ CFU and were examined 24 h post-injection. SEZ infection triggered overt jejunal hemorrhage and upregulation of IL-1β, IL-6, and TNF-α mRNA, confirming local inflammation, with a concurrent marked reduction in both mRNA and protein levels of IRAK M in the jejunum, hinting at its possible role. We next used IRAK-M knockout (IRAK-M^−/−^) mice and found that, compared with the WT+SEZ group, the knockout group displayed more severe jejunal hemorrhage, histopathological scores, and neutrophil infiltration, along with a higher bacterial burden and further elevated pro-inflammatory cytokines and reduced anti-inflammatory cytokine expression. Mechanistically, IRAK-M deficiency in the jejunum further increased the mRNA expression of MyD88, TRIF, TRAF6, IKKα+β, NF-κBp50, and NF-κBp65 and decreased IκBα mRNA compared with the WT+SEZ group. These findings were paralleled at the protein level, as demonstrated by Western blotting for MyD88, TRAF6, and IKKα+β, and by immunofluorescence in peritoneal macrophages. Importantly, IRAK-M deficiency drove a shift toward the M1 macrophage phenotype in both jejunal tissues and peritoneal macrophages, characterized by upregulation of CD16, CD32, CD80, CD86, iNOS, and MHC II, and downregulation of the M2-associated markers Arg1 and CD206. These findings reveal that SEZ infection causes jejunal inflammation and that IRAK-M knockout worsens this condition, pointing to a role for IRAK-M in regulating inflammatory readouts in this model.

## 1. Introduction

*Streptococcus equi* subsp. *zooepidemicus* (SEZ) is a zoonotic pathogen that poses a persistent public health threat [1,2]. As a Lancefield group C β-hemolytic streptococcus, SEZ exhibits a broad mammalian host range, causing severe diseases in livestock and companion animals while often remaining asymptomatic in equines [3,4]. Although human infections are infrequent, they carry a heightened risk of grave outcomes, including meningitis, septicemia, and endocarditis [5]. These severe systemic manifestations are frequently linked to defined exposure routes, such as direct contact with infected animals or consumption of contaminated unpasteurized dairy products, as evidenced by documented outbreaks in Finland [6], Spain [7], and Italy [8]. While these transmission pathways underscore the zoonotic potential of SEZ, the pathogenesis following systemic dissemination remains poorly understood. Notably, we observed in our preliminary experiments that intraperitoneal SEZ infection in mice caused evident jejunal pathology; however, the specific inflammatory responses and mucosal injury elicited during systemic SEZ infection remain largely unexplored.

Macrophages act as sentinel cells in the host response to bacterial infection, initiating inflammatory programs upon recognition of conserved microbial motifs via pattern recognition receptors [9]. Depending on the nature of the stimulus, macrophages can adopt a pro-inflammatory M1 phenotype, characterized by elevated surface expression of MHC-II, CD80, and CD86, along with robust production of inflammatory cytokines [9,10]. This polarization process is largely governed by the NF-κB signaling axis, which serves as a central hub linking microbial detection to the transcriptional regulation of immune effector genes [9]. Accordingly, various bacterial species have evolved strategies to exploit or subvert NF-κB activity, thereby reprogramming macrophage polarization in ways that favor their own persistence. For example, *enterococcal* infection has been reported to drive M1 polarization through NF-κB activation [11], and *Salmonella* indirectly induces macrophages toward an M2-like immunosuppressive phenotype, marked by Arg1 and CD206, thereby promoting its intracellular survival [12]. Despite these findings in other pathogens, it remains largely unknown whether SEZ infection similarly modulates macrophage phenotype and NF-κB signaling within the intestinal mucosa.

IRAK-M (interleukin-1 receptor-associated kinase M) is distinguished as a unique member of the IRAK family, serving as a pivotal negative regulator of Toll-like receptor (TLR) signaling [13]. Disruption of IRAK-M function has been linked to a range of inflammatory conditions. Studies using IRAK-M-deficient mice have demonstrated that loss of IRAK-M amplifies inflammatory responses in experimental models of sepsis and colitis, with concomitant hyperactivation of NF-κB and elevated secretion of pro-inflammatory mediators [14,15]. In contrast, upregulation of IRAK-M has been documented in certain malignancies and persistent infections, where it contributes to immune evasion and sustained pathogen colonization [16]. Collectively, the above observations underscore the dual nature of IRAK-M in maintaining the equilibrium between protective immunity and inflammatory pathology. Nevertheless, despite its well-recognized role in constraining TLR-driven inflammation, the potential involvement of IRAK-M in modulating macrophage phenotype and NF-κB signaling during specific bacterial infections remains poorly defined.

In the present study, we hypothesized that IRAK-M modulates intestinal inflammation in SEZ-infected mice by regulating NF-κB signaling and macrophage phenotype. Our results demonstrated that SEZ infection elicited jejunal inflammation and concurrently downregulated IRAK-M expression in mice. Furthermore, IRAK-M deficiency exacerbated the inflammatory response, modulated NF-κB signaling, and skewed macrophages toward the M1 phenotype. These data suggest that IRAK-M contributes to the regulation of inflammatory responses in this SEZ infection model.

## 2. Materials and Methods

### 2.1. Mice

Male wild-type (WT) C57BL/6 mice (6–8 weeks old) were obtained from Guangdong Yaokang Biotechnology Co., Ltd. (Guangzhou, China), and IRAK-M knockout (KO) mice on the C57BL/6 background were purchased from The Jackson Laboratory (007016; West Grove, PA, USA). The KO mice were backcrossed to WT C57BL/6 mice prior to use to minimize genetic and microbiota variability, and genotypes were confirmed by PCR using tail-derived DNA. All mice were co-housed in mixed-genotype cages for at least 2 weeks before experimentation to harmonize gut microbiota. Animals were housed under specific pathogen-free conditions (20 °C ± 3 °C, 60% ± 5% relative humidity) with food and water ad libitum. The study was carried out following the guidelines approved by the Foshan University Experimental Animal Ethics Committee (Approval No. FOSU20240107001). All experimental procedures were designed to reduce pain and to use the minimum number of animals necessary for statistically valid results.

### 2.2. Bacterial Strains

The SEZ strain C55138, sourced from the China Institute of Veterinary Drug Control (Beijing, China), was cultured at 37 °C on tryptic soy agar (TSA; Oxoid, Basingstoke, UK) or in tryptic soy broth (TSB; Oxoid, Basingstoke, UK) with 5% newborn calf serum (Hyclone, Logan, UT, USA). After growth to the logarithmic phase, the bacteria were harvested via centrifugation (3500× *g*, 10 min), washed in sterile phosphate-buffered saline (PBS), and diluted in series for subsequent in vivo experiments.

### 2.3. Mice Challenged with SEZ

Mice were divided into three groups: the control group (CON), the SEZ-infected group (WT+SEZ), and the IRAK-M knockout plus SEZ-infected group (IRAK-M^−/−^+SEZ), with 12 mice in each group. Mice in the SEZ-infected groups were intraperitoneally injected with SEZ suspension at a dose of 2 × 10^7^ CFU per mouse [1]. At 24 h post inoculation, after excluding mice that had died or experienced procedural failure, the remaining animals were anesthetized in a sealed chamber with 4–5% isoflurane in oxygen, resulting in loss of spontaneous activity and righting reflex within 1–2 min. After the toe-pinch test confirmed deep anesthesia, mice were immediately euthanized by cervical dislocation (performed by trained staff). Jejunal tissues were collected promptly and cut into two parts: one fixed in 4% paraformaldehyde for histology, the other snap-frozen at −80 °C for later assays.

### 2.4. Hematoxylin and Eosin Staining

For histological evaluation, jejunal tissues from six mice per group were processed according to standard protocols. Briefly, specimens were fixed in 4% paraformaldehyde for 48 h, dehydrated through a graded ethanol series, cleared, and embedded in paraffin. Sections were cut at 8 μm thickness, mounted onto glass slides, and stained with hematoxylin and eosin (H&E) for morphological examination. Stained slides were visualized under a Nikon Ni-U microscope (Nikon Corporation, Tokyo, Japan) at both 4× and 40× magnifications to assess jejunal architecture and inflammatory changes.

For semi-quantitative assessment, five randomly selected non-adjacent fields per section were examined from three non-contiguous sections per animal, yielding a total of 15 fields per mouse. All slides were coded with random numbers, and histopathological scoring was performed independently by two investigators who were blinded to the experimental groups. A four-criterion scoring system ranging from 0 (normal) to 3 (severe) was adopted, evaluating: villus integrity (intact to total atrophy), epithelial loss (none to >50% denudation or ulceration), inflammatory cell infiltration (≤5 to >30 cells per high-power field or crypt abscess), and crypt architecture (regular to completely destroyed). The final score for each animal was calculated as the sum of the scores for all four criteria (total possible score: 0–12), averaged across the 15 fields examined. The mean values from the two scorers were used for subsequent statistical analysis [17].

### 2.5. Bacterial Load

For each group, 12 mice were used with three replicates per mouse, and jejunal tissues were aseptically collected, weighed, and homogenized in sterile PBS at a ratio of 100 mg tissue per 1 mL PBS using a tissue grinder. The homogenates were serially diluted 10-fold in PBS, and 100 μL aliquots of appropriate dilutions were spread onto TSA plates. After incubation at 37 °C for 24 h, the number of CFU was counted, and the bacterial burden was calculated as log_10_ CFU per gram of jejunal tissue.

### 2.6. RNA Extraction and Quantitative Real-Time PCR

For quantitative real-time PCR (qRT-PCR) analysis, total RNA was isolated from jejunal tissues obtained from six mice per experimental group using the commercial RNA extraction kit DP424 (TianGen, Beijing, China), with all procedures carried out in strict accordance with the manufacturer’s protocol. The purity and integrity of the extracted RNA were evaluated by spectrophotometric measurement of the A_260_/A_280_ absorbance ratio, and only specimens yielding ratios falling within the range of 1.8 to 2.0 were retained for downstream applications. First-strand cDNA was synthesized from 1 µg of purified RNA per reaction using the HiScript II First Strand cDNA Synthesis Kit (Vazyme Biotech Co., Ltd., Nanjing, China), following the recommended protocol. The cDNA products were subsequently diluted to a final working concentration of 10 ng/µL and served as templates for qRT-PCR amplifications, which were performed with 2× UltraSYBR Green qPCR Master Mix (CW0957M, CWBIO, Taizhou, China) and gene-specific oligonucleotide primers. Specificity of each primer pair was verified through melting curve analysis, which produced a single distinct peak for every amplicon, while amplification efficiency was assessed using five-point serial dilution standard curves; primer pairs were considered acceptable only when efficiency values ranged between 90% and 110% and the corresponding R^2^ coefficients exceeded 0.98. The thermal cycling regimen comprised an initial denaturation step at 95 °C for 10 min, followed by 40 cycles of 94 °C for 30 s, 60 °C for 30 s, and 72 °C for 30 s. At the conclusion of each run, a melting curve analysis was conducted over a temperature gradient of 65–95 °C with 0.5 °C increments to further confirm the specificity of the amplified products. Relative transcript abundances were determined using the 2^−ΔΔCt^ method, with GAPDH serving as the endogenous reference gene. The stability of GAPDH expression across the various experimental conditions was validated in preliminary experiments, and its reliability as a reference gene in this intestinal inflammation model is well supported by previous studies. A complete list of primer sequences is provided in Table 1, and all qRT-PCR assays were conducted in triplicate [18].

### 2.7. Western Blotting (WB)

Protein extracts were obtained from jejunal tissues of four mice per group (with three replicates per mouse) using Mammalian Protein Extraction Reagent (Solarbio, Beijing, China), and the total protein content of each sample was determined via the BCA assay. Equal amounts of protein were separated by electrophoresis on 10% SDS-polyacrylamide gels and then electrotransferred onto PVDF membranes. The membranes were subsequently blocked and probed with specific primary antibodies (Table 2), followed by incubation with an HRP-conjugated secondary antibody. Immunoreactive signals were visualized using the BIO-RAD ChemiDoc™ MP imaging system (Bio-Rad Laboratories, Inc., Hercules, CA, USA). Densitometric analysis of the resulting bands was performed, and the relative expression levels were calculated after normalization to β-actin as the internal loading control.

### 2.8. Isolation and Immunofluorescence Assay of Peritoneal Macrophages

Peritoneal cells were collected by lavage from six randomly selected mice per group. Following surface disinfection with 75% ethanol, each mouse was placed in a sterile laminar flow hood, and 5 mL of ice-cold PBS was slowly injected into the peritoneal cavity. The abdominal wall was gently massaged for approximately 3 min to dislodge resident peritoneal cells. The lavage fluid was then aspirated, and this rinsing procedure was repeated three times. The pooled cell suspension was centrifuged at 800 rpm for 5 min at 4 °C. After discarding the supernatant, the cell pellet was treated with erythrocyte lysis buffer for 5 min, resuspended in PBS, and washed twice by centrifugation. The final pellet was resuspended in DMEM supplemented with 10% fetal bovine serum (FBS) at a density of 2 × 10^5^ cells/mL, seeded into culture vessels, and incubated at 37 °C in 5% CO_2_ for 3 h to allow adherence. Non-adherent cells were removed by two gentle PBS washes, and the remaining adherent cells were used as a macrophage-enriched population for subsequent experiments. This adherence-based enrichment method is widely accepted for obtaining primary peritoneal macrophages and yields high purity suitable for functional assays [18].

For immunofluorescence staining, three replicates were performed per mouse, and these adherent cells were permeabilized with 1% Triton X-100 at 37 °C to allow intracellular antibody access, and then blocked with 5% goat serum to minimize background labeling. Primary antibodies (Table 3) were applied and incubated overnight at 4 °C. After thorough washing, the cells were incubated with Alexa Fluor 488-conjugated goat anti-rabbit IgG secondary antibody (1:100; A11034, Invitrogen, Waltham, MA, USA) at 4 °C, followed by nuclear counterstaining with DAPI (0100-20; SouthernBiotech, Birmingham, AL, USA) at room temperature. Negative controls were prepared by substituting PBS for the primary antibody. Fluorescent images were captured using an inverted fluorescence microscope (T00423, ZEISS, Baden-Württemberg, Germany), with five random fields selected per sample and all samples randomly coded for analysis by two independent investigators completely blinded to group allocation.

### 2.9. Statistical Analysis

All statistical analyses were conducted using GraphPad Prism version 9 (GraphPad Software, San Diego, CA, USA). Data normality and variance homogeneity were assessed via the Shapiro–Wilk and Brown–Forsythe tests, respectively. For normally distributed continuous variables, comparisons between two groups were performed using unpaired two-tailed Student’s *t*-tests. To address multiple testing across cytokine, signaling, and macrophage marker datasets, *p*-values were adjusted using the Holm–Šidák method to control the family-wise error rate. Histological scores, being ordinal data, were analyzed using the Mann–Whitney *U* test rather than parametric methods. Results are expressed as mean ± standard deviation (SD) derived from a minimum of three independent experiments. Statistical significance was defined as *p* < 0.05, with *p* < 0.01 and *p* < 0.001 denoting higher levels of significance.

## 3. Results

### 3.1. Infection with SEZ Results in Jejunal Inflammation and Suppresses IRAK-M Expression in Mice

**Figure 1:**
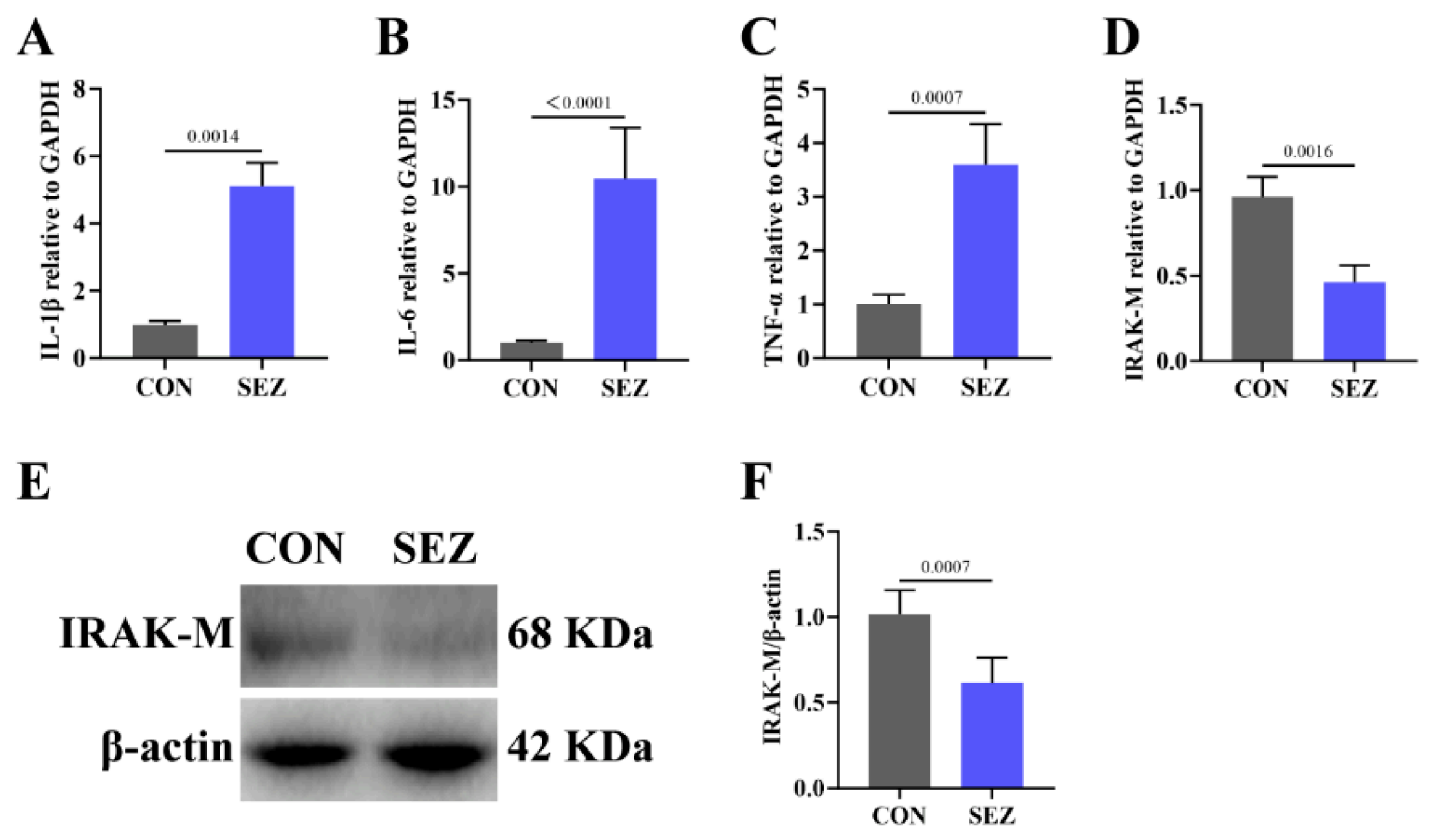
SEZ infection leads to inflammation in the mouse jejunum and concurrently suppresses IRAK-M expression. (**A**–**D**) qRT-PCR was employed to quantify the mRNA levels of IL-1β, IL-6, TNF-α, and IRAK-M in jejunal tissues. *n* = 6. (**E**,**F**) The protein abundance of IRAK-M in jejunal tissues was assessed via WB. *n* = 4. For qRT-PCR, GAPDH served as the internal reference, and relative fold changes were calculated using the 2^−ΔΔCt^ method. For WB, β-actin was employed as the loading control. All data are expressed as mean ± SD.

Mice received an intraperitoneal injection of SEZ suspension at 2 × 10^7^ CFU per mouse and were euthanized 24 h post-infection. At necropsy, the jejunum of SEZ-infected mice displayed gross hemorrhagic lesions compared with the CON group. To verify whether these macroscopic changes were associated with inflammation, we examined the expression of inflammatory cytokines in the jejunum. The results showed that the mRNA levels of the pro-inflammatory cytokines IL-1β, IL-6, and TNF-α were significantly elevated in the infected mice relative to controls, confirming that SEZ infection effectively induced jejunal inflammation (Figure 1A–C). Given that IRAK-M is a critical negative regulator of inflammatory signaling, we next assessed its expression in jejunal tissues. Both qRT-PCR and WB analyses consistently revealed that SEZ infection markedly downregulated IRAK-M at both the transcriptional and protein levels (Figure 1D–F). These findings indicate that SEZ infection triggers jejunal inflammation and that IRAK-M may be involved in this pathological process.

### 3.2. IRAK-M Deficiency Leads to Markedly Aggravated Jejunal Tissue Damage and Inflammation

**Figure 2:**
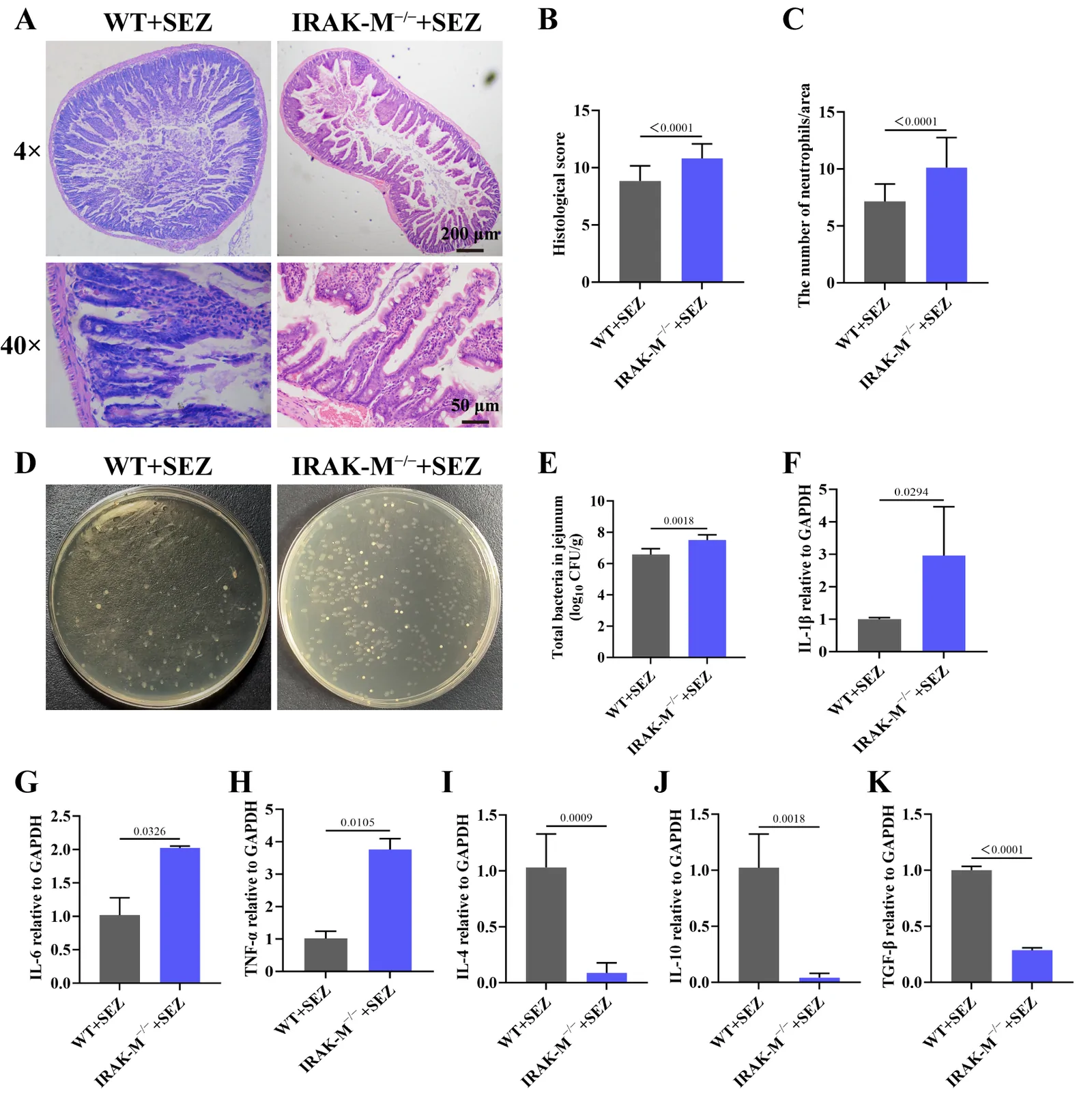
Knockout of IRAK-M worsens SEZ-triggered jejunal injury and inflammatory responses in mice. (**A**) Representative H&E-stained jejunal sections are shown at 10× (scale bar, 200 μm) and 40× (scale bar, 50 μm) magnifications. (**B**,**C**) Histological scoring and neutrophil counts per microscopic field were assessed on the H&E-stained sections. *n* = 6. (**D**,**E**) Bacterial colonization in jejunal tissues across the experimental groups was measured. *n* = 12. (**F**–**K**) qRT-PCR was employed to quantify the mRNA levels of inflammatory cytokines in jejunal tissues, with GAPDH serving as the reference gene. *n* = 6. Relative expression was calculated by the 2^−ΔΔCt^ method, normalized to the WT+SEZ group. All data are expressed as mean ± SD.

To investigate the potential role of IRAK-M in SEZ-induced jejunal inflammation, we generated IRAK-M knockout mice. Histological examination by H&E staining showed that IRAK-M^−/−^+SEZ mice developed markedly more severe jejunal mucosal injury than WT+SEZ mice, characterized by extensive epithelial shedding, dense infiltration of inflammatory cells into the submucosa and lamina propria, and multifocal hemorrhage (Figure 2A). Consistently, histopathological scores and neutrophil infiltration counts were both significantly elevated in the IRAK-M^−/−^+SEZ group compared with the WT+SEZ group (Figure 2B,C). Quantification of viable bacteria by plate counting from jejunal tissue homogenates revealed a marked increase in total cultivable bacterial burden in IRAK-M^−/−^ mice following SEZ infection, indicating enhanced bacterial outgrowth within the jejunal wall (Figure 2D,E). Moreover, qRT-PCR analysis indicated that the mRNA levels of pro-inflammatory cytokines IL-1β, IL-6, and TNF-α were markedly upregulated, whereas those of anti-inflammatory cytokines IL-4, IL-10, and TGF-β were significantly downregulated in the knockout group (Figure 2F–K). Taken together, these findings demonstrate that IRAK-M deficiency potently exacerbates SEZ infection-induced jejunal inflammation and mucosal injury.

### 3.3. IRAK-M Gene Deletion Further Upregulates NF-κB-Related Molecules in the Jejunum of SEZ-Infected Mice

**Figure 3:**
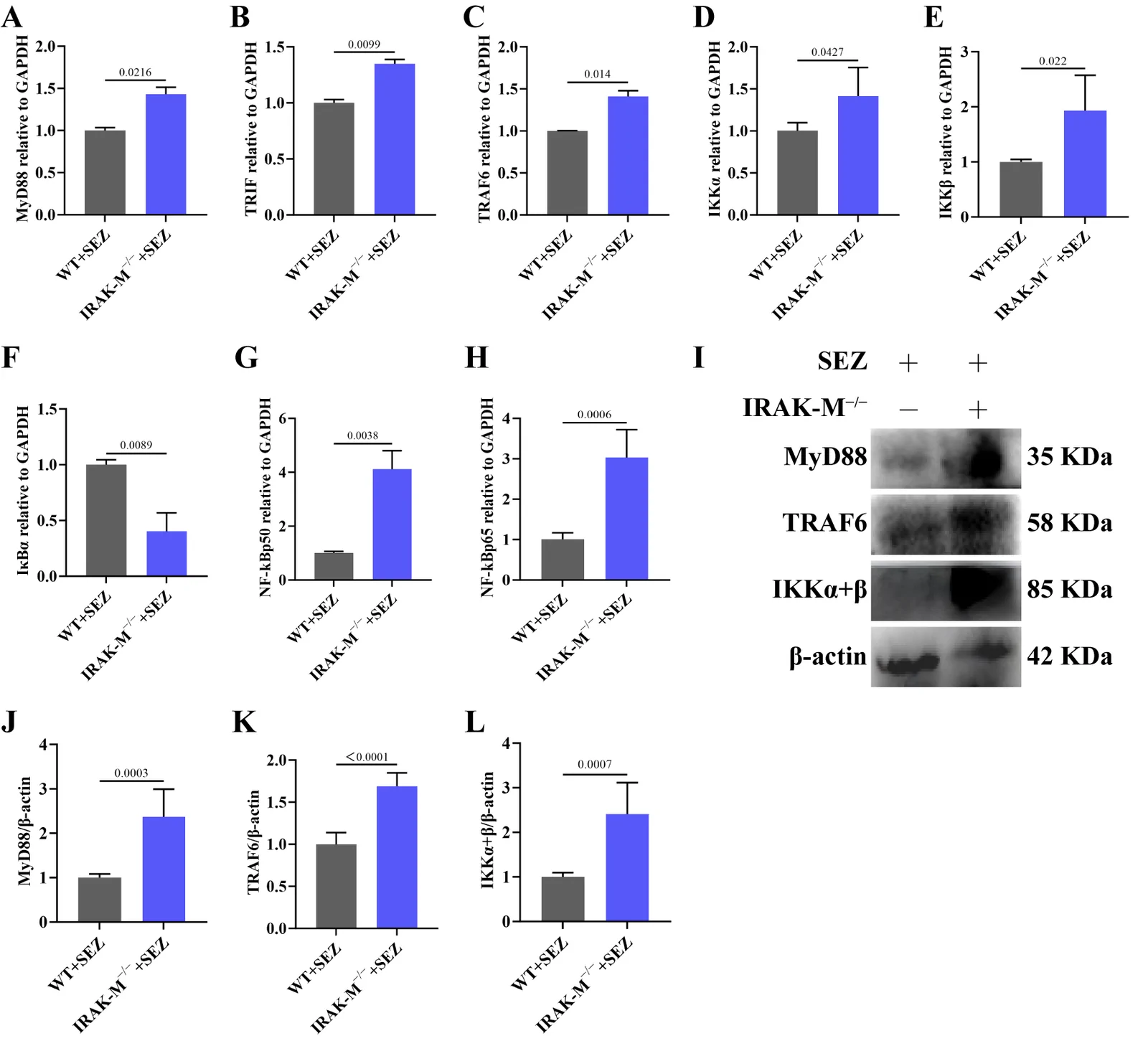
IRAK-M knockout further changes NF-κB signaling in the jejunum of SEZ-infected mice. (**A**–**H**) The mRNA transcript levels of MyD88, TRIF, TRAF6, IKKα, IKKβ, IκBα, NF-κBp50, and NF-κBp65 in jejunal tissues were determined by qRT-PCR. *n* = 6. (**I**–**L**) The protein abundance of MyD88, TRAF6, and IKKα+β in jejunal tissues was assessed via WB. *n* = 4. For qRT-PCR, GAPDH served as the internal reference, and relative fold changes were calculated using the 2^−ΔΔCt^ method. For WB, β-actin was employed as the loading control. All data are expressed as mean ± SD.

Given the critical role of the NF-κB signaling pathway in inflammatory responses, we next examined the expression of its key components. qRT-PCR analysis revealed that the mRNA levels of MyD88, TRIF, TRAF6, IKKα, IKKβ, NF-κBp50, and NF-κBp65 in the jejunum were significantly upregulated, while the expression of IκBα was decreased, in IRAK-M^−/−^+SEZ mice compared with WT+SEZ mice (Figure 3A–H). Consistently, WB analysis further confirmed that IRAK-M deficiency also markedly increased the protein levels of MyD88, TRAF6, and IKKα+β in the jejunal tissues of SEZ-infected mice (Figure 3I–L). Together, these findings at both the mRNA and protein levels indicate that loss of IRAK-M significantly alters NF-κB pathway components in the jejunum following SEZ infection.

### 3.4. Absence of IRAK-M Upregulates NF-κB-Related Proteins in Peritoneal Macrophages

**Figure 4:**
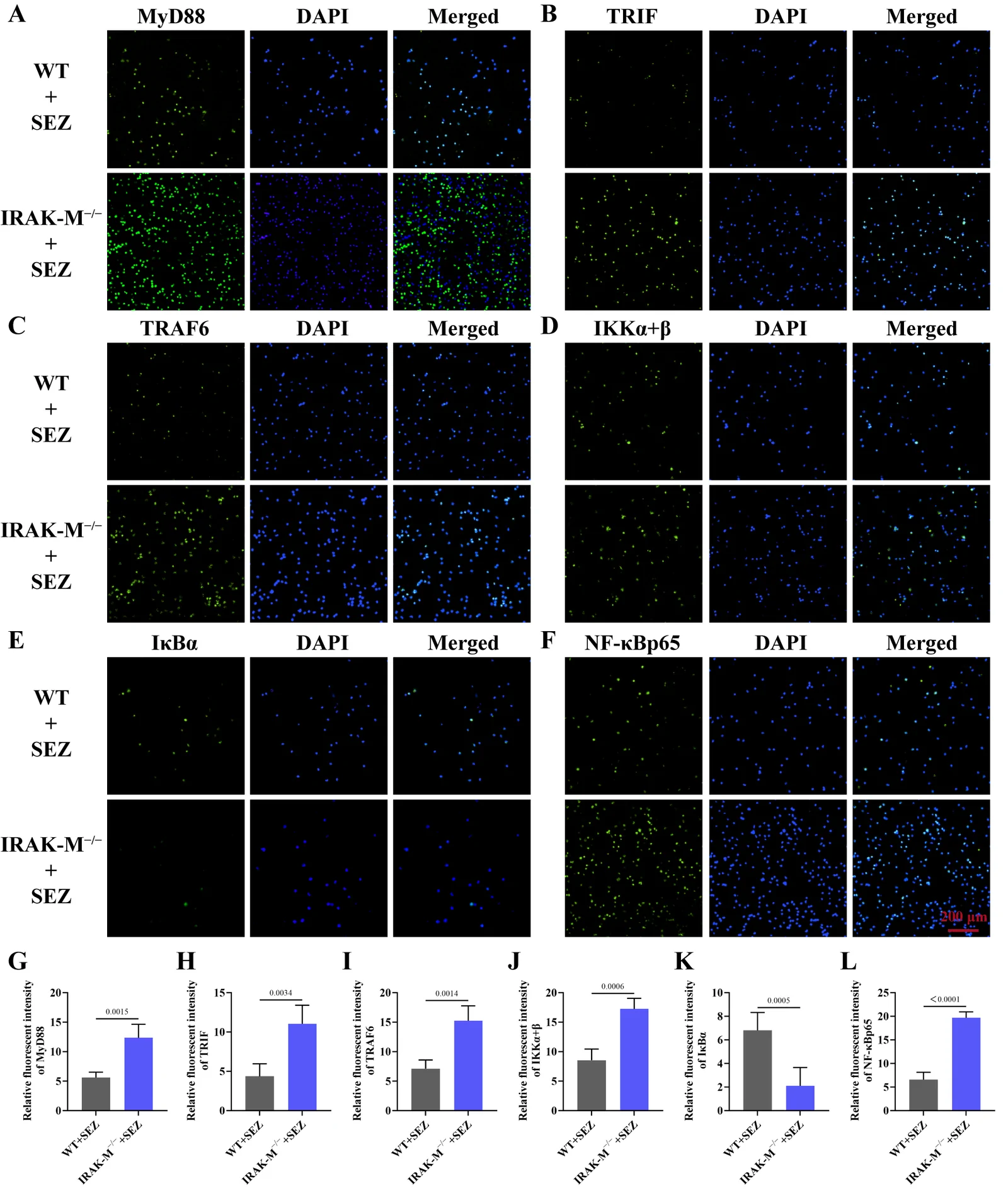
IRAK-M ablation leads to increased NF-κB signaling in peritoneal macrophages following SEZ infection. (**A**–**L**) The expression of MyD88, TRIF, TRAF6, IKKα+β, IκBα, and NF-κBp65 in peritoneal macrophages was assessed by immunofluorescence staining and relative fluorescence intensity quantification. *n* = 6. All data are expressed as mean ± SD.

To further verify the impact of IRAK-M deficiency on NF-κB signaling, peritoneal macrophages were isolated from each group of mice and subjected to immunofluorescence staining. In the WT+SEZ group, clear positive fluorescence signals for MyD88, TRIF, TRAF6, IKKα+β, and NF-κBp65 were observed within macrophages, whereas the fluorescence intensity of IκBα was relatively weak (Figure 4A–F). In contrast, macrophages from IRAK-M^−/−^+SEZ mice displayed notably enhanced fluorescence intensities for all the above-mentioned proteins, along with a marked reduction in IκBα fluorescence, as also confirmed by relative fluorescence intensity quantification (Figure 4A–L). These findings indicate that loss of IRAK-M leads to significant changes in NF-κB pathway components in macrophages.

### 3.5. IRAK-M Deficiency Skews Jejunal Macrophages Toward the M1 Phenotype in SEZ-Infected Mice

**Figure 5:**
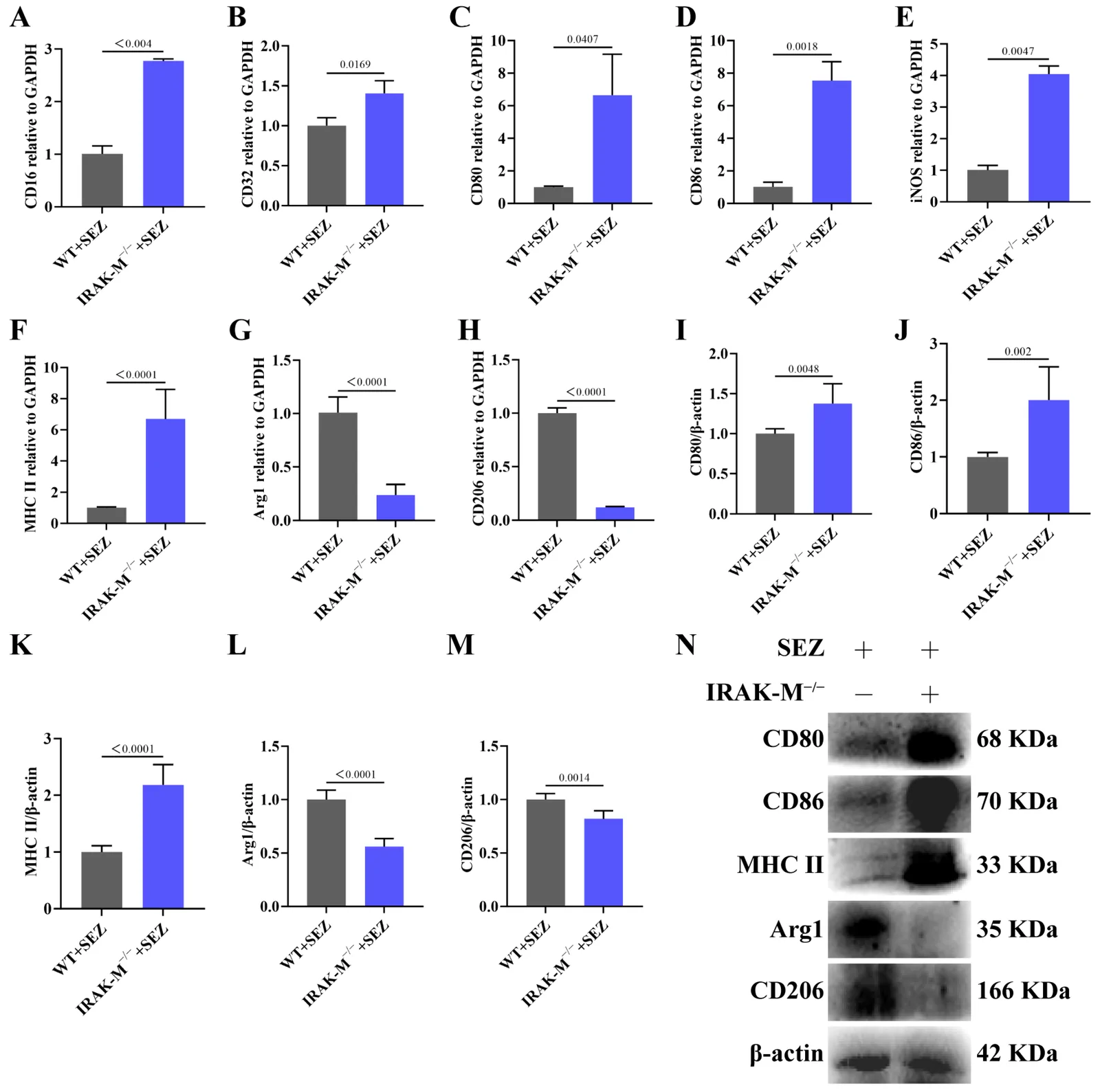
IRAK-M ablation skews jejunal macrophages toward the M1 phenotype in SEZ-infected mice. (**A**–**H**) qRT-PCR data show the mRNA levels of macrophage-associated markers (CD16, CD32, CD80, CD86, iNOS, MHC II, Arg1, and CD206) in jejunal tissues. *n* = 6. (**I**–**N**) WB analysis displays the protein abundance of CD80, CD86, MHC II, Arg1, and CD206 in the same tissues. *n* = 4. Gene expression was normalized to GAPDH, and fold changes were calculated using the 2^−ΔΔCt^ method. For protein detection, β-actin was used as the loading control. Data represent mean ± SD.

Macrophages play a pivotal role in inflammatory responses. To further elucidate the impact of IRAK-M deficiency on macrophage phenotype, we assessed the transcriptional levels of polarization-related genes by qRT-PCR. Compared with the WT+SEZ group, the IRAK-M^−/−^+SEZ group exhibited significantly upregulated mRNA expression of M1-type markers CD16, CD32, CD80, CD86, iNOS, and MHC II in the jejunum, whereas the mRNA levels of M2-type markers Arg1 and CD206 were markedly downregulated (Figure 5A–H). Consistently, WB analysis further confirmed that protein levels of CD80, CD86, and MHC II were significantly increased in the IRAK-M-deficient group, while those of Arg1 and CD206 were decreased, relative to the WT+SEZ group (Figure 5I–N). Collectively, these findings suggest that IRAK-M deficiency promotes M1 phenotype of jejunal macrophages in SEZ-infected mice.

### 3.6. IRAK-M Knockout Skews Peritoneal Macrophages Toward the M1 Phenotype

**Figure 6:**
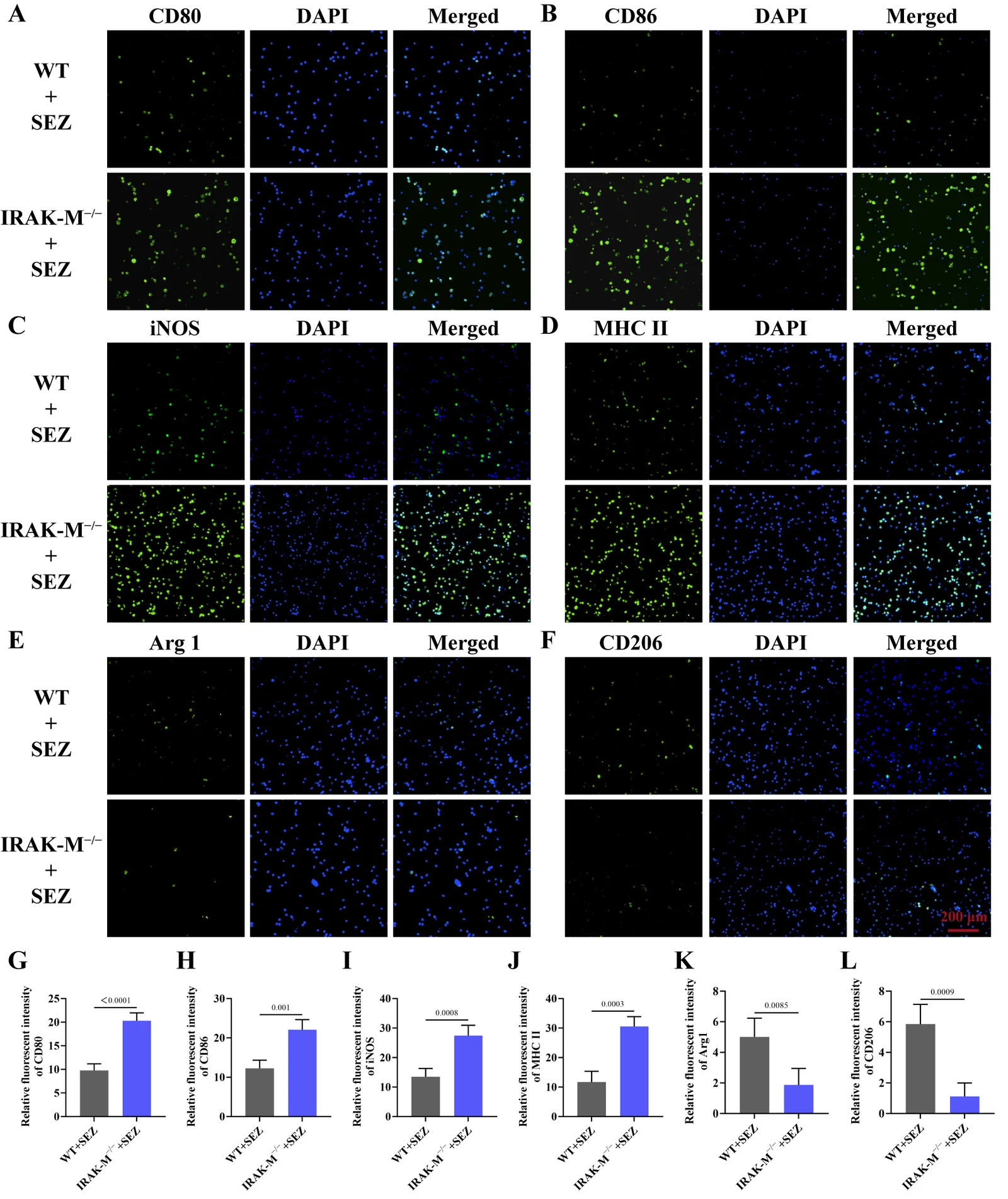
Knockout of IRAK-M drives peritoneal macrophages of SEZ-challenged mice toward the M1 phenotype. (**A**–**L**) The expression of CD80, CD86, iNOS, MHC II, Arg1, and CD206 in peritoneal macrophages was assessed by immunofluorescence staining and relative fluorescence intensity quantification. *n* = 6. All data are expressed as mean ± SD.

Immunofluorescence staining was performed on peritoneal macrophages from each group, and fluorescence microscopy revealed that M1-type markers CD80, CD86, iNOS, and MHC II exhibited clear positive signals in the WT+SEZ group (Figure 6A–D), whereas M2-type markers Arg1 and CD206 showed relatively weak fluorescence intensities (Figure 6E,F). Compared with the WT+SEZ group, the IRAK-M^−/−^+SEZ group displayed enhanced fluorescence intensities of M1-type markers and diminished fluorescence intensities of M2-type markers, which was further corroborated by the quantitative analysis of relative fluorescence intensity (Figure 6A–L). These results further indicate that loss of IRAK-M promotes macrophage-associated marker expression toward the M1 phenotype.

## 4. Discussion

Our findings showed that SEZ infection induced inflammation in the murine jejunum and simultaneously reduced IRAK-M expression. Importantly, loss of IRAK-M further potentiated the inflammatory response, elevated expression of NF-κB-related components, and drove macrophages toward a pro-inflammatory M1 phenotype. These observations indicate that IRAK-M contributes to the regulation of inflammatory responses in SEZ-associated enteritis.

Our observation that IRAK-M deficiency markedly exacerbates SEZ-induced jejunal mucosal damage, accompanied by increased bacterial burden, and a pronounced shift toward a pro-inflammatory cytokine profile (upregulated IL-1β, IL-6, TNF-α and downregulated IL-4, IL-10, TGF-β), is corroborated by the well-documented function of IRAK-M as a negative regulator of TLR-mediated inflammatory responses [13,19]. In the context of intestinal inflammation, accumulating evidence indicates that IRAK-M deficiency worsens the severity of colitis [14,18,20,21,22], concurrent with augmented production of pro-inflammatory mediators and intensified tissue destruction, a pattern that closely parallels our observation of aggravated mucosal injury and increased IL-1β, IL-6, and TNF-α in IRAK-M^−/−^ mice. In contrast to earlier studies showing that IRAK-M deficiency does not increase host bacterial loads [23], we found that IRAK-M-knockout mice had markedly more bacteria in the jejunum, implying a potential impairment of antimicrobial defense upon IRAK-M deletion. The concomitant reduction in anti-inflammatory cytokine expression, including IL-4, IL-10, and TGF-β, reinforces the concept that IRAK-M deficiency shifts the immune equilibrium toward a persistently pro-inflammatory milieu, an alteration that has been similarly documented across various IRAK-M-related intestinal pathological conditions. Importantly, although IRAK-M has been extensively characterized in models of sterile inflammation and chemically induced colitis, the current work presents, to our knowledge, the first evidence that IRAK-M deficiency exacerbates jejunal mucosal injury and inflammation in the setting of SEZ infection, a finding of particular relevance given the increasing appreciation of SEZ as a zoonotic pathogen with enteric disease potential.

Our findings indicate that IRAK-M deficiency promotes the upregulation of NF-κB-associated molecules and skews jejunal macrophages toward an M1 phenotype in SEZ-infected mice. By stabilizing the MyD88–IRAK4–IRAK1/2 assembly in an inactive conformation, IRAK-M prevents downstream TRAF6 ubiquitination and subsequent IKK-mediated IκBα degradation, ultimately restraining NF-κB nuclear translocation [19]. Consistent with that mechanistic framework, our qRT-PCR and WB analyses revealed that IRAK-M^−/−^ mice infected with SEZ exhibited markedly elevated transcript and protein levels of MyD88, TRIF, TRAF6, IKKα, IKKβ, NF-κBp50, and NF-κBp65 in jejunal tissues, accompanied by a conspicuous reduction in IκBα expression. Such molecular changes are reminiscent of previous findings that IRAK-M deficiency exacerbated dopaminergic neuronal damage in a sub-acute Parkinson’s disease mouse model through amplified NF-κB-driven neuroinflammation [24], as well as recent work demonstrating that airway-expressed IRAK-M modulates JNK and NF-κB signaling to govern the magnitude of inflammatory responses during nontypeable *Haemophilus influenzae* infection [25]. Importantly, our immunofluorescence data obtained from peritoneal macrophages further showed that IRAK-M^−/−^ macrophages exhibited enhanced fluorescence intensities for MyD88, TRIF, TRAF6, IKKα+β, and NF-κBp65, while IκBα fluorescence was reciprocally decreased relative to WT cells, indicating that the changes in NF-κB-related molecules observed at the tissue level were also detectable in macrophages.

The convergence of NF-κB hyperactivation and macrophage polarization represents a biologically coherent and functionally significant finding. It is well established that phosphorylated p65, upon nuclear translocation, directly transcribes M1-associated genes including iNOS, TNF-α, IL-1β, and IL-6, while simultaneously suppressing M2-associated transcriptional programs [26,27]. In our work, the transcriptional upregulation of M1 markers (CD16, CD32, CD80, CD86, iNOS, and MHC II) and the concurrent downregulation of M2 markers (Arg1 and CD206) in the jejunum of IRAK-M^−/−^+SEZ mice, further validated at the protein level by WB and immunofluorescence, suggest a shift toward a pro-inflammatory macrophage-associated response in the jejunal microenvironment upon IRAK-M deficiency. These observations are in line with a recent report showing that IL-7R deficiency attenuated NF-κB signaling and consequently reduced M1 macrophage polarization in an abdominal aortic aneurysm model [28], as well as a study demonstrating that CCL8 induces M1 polarization through NF-κBp65 activation in ovarian cancer [29]. However, what distinguishes our work from these investigations is the infectious etiology and the jejunal locale. To date, the interplay between IRAK-M, NF-κB, and macrophage polarization has been predominantly examined in sterile inflammatory conditions, tumor microenvironments, or pulmonary infection models [18,22,25,30,31,32]. The jejunal mucosa, with its unique immunological architecture comprising lamina propria macrophages, and a constant flux of luminal microbial stimuli, presents a fundamentally different immunological landscape. Our finding that SEZ, a pathogen traditionally associated with meningitis, septicemia, and reproductive tract infections in swine and equids, can elicit a robust IRAK-M–NF-κB–macrophage phenotype axis in the jejunum constitutes a novel and intriguing observation that broadens the pathophysiological spectrum of the zoonotic organism.

Nevertheless, several limitations must be acknowledged. The absence of uninfected WT and IRAK-M^−/−^ controls precludes definitive attribution of observed differences to infection-specific effects. The intraperitoneal challenge model, while established for systemic SEZ infection, may not fully recapitulate the natural enteral route and the mucosal immune dynamics it engages. Potential confounding by vendor source, genetic background drift, or microbiota composition cannot be excluded. As our conclusions are based mainly on male mice, caution is needed when extrapolating to females, and future validation in female mice is warranted. Neutrophils were quantified based on H&E morphology alone, without molecular markers such as Ly6G or MPO, potentially limiting the precision of our findings. Furthermore, although our data establish a correlative link among IRAK-M loss, NF-κB pathway alterations, and M1 polarization, definitive proof of NF-κB activation and causality would require rescue experiments such as pharmacological inhibition of NF-κB in IRAK-M^−/−^ mice. The contribution of other IRAK-M-regulated pathways, such as MAPK/JNK and IRF3/7 signaling, was not interrogated and may synergize with or diverge from the NF-κB axis. Additionally, peritoneal macrophages analyzed here differ from jejunal tissue-resident macrophages in origin and niche, and thus may not fully reflect jejunal macrophage responses in situ.

## 5. Conclusions

Our findings revealed that SEZ infection elicited jejunal inflammation and concomitantly downregulated IRAK-M expression in mice. Furthermore, loss of IRAK-M aggravated the inflammatory response, upregulated NF-κB-related components, and promoted the expression of macrophage-associated markers toward the M1 phenotype. Collectively, these observations suggest that IRAK-M contributes to the regulation of inflammatory responses in this SEZ infection model.

## Figures and Tables

**Table 1:** Specific primers used in qRT-PCR analysis.

Gene	Direction	Primer Sequence (5′→3′)
IL-1β	Forward Reverse	GCTGCTTCCAAACCTTTGAC AGCTTCTCCACAGCCACAAT
IL-4	Forward Reverse	CCCCAGCTAGTTGTCATCCTG CAAGTGATTTTTGTCGCATCCG
IL-6	Forward Reverse	TAGTCCTTCCTACCCCAATTTCC TTGGTCCTTAGCCACTCCTTC
IL-10	Forward Reverse	CCAGTTTTACCTGGTAGAAGTGATG TGTCTAGGTCCTGGAGTCCAGCAGACTCAA
TNF-α	Forward Reverse	CCGATGGGTTGTACCTTGTC AGATAGCAAATCGGCTGACG
TGF-β	Forward Reverse	CCAGATCCTGTCCAAACTAAGG CTCTTTAGCATAGTAGTCCGCT
MyD88	Forward Reverse	GCTAGAGCTGCTGGCCTTGTTAG TCTCGGACTCCTGGTTCTGCTG
TRIF	Forward Reverse	CCACGTCCTACACGGAAGAT AACAGCATCTGCAGCTACCA
TRAF6	Forward Reverse	TACGATGTGGAGTTTGACCCA CACTGCTTCCCGTAAAGCCAT
IKKα	Forward Reverse	GAGAGCGATGGTGCCATGAA CCAGAACAGTACTCCATTGCCAGA
IKKβ	Forward Reverse	GCCTTATGAACGAGGACGAG CTGTCTGGGCTTCCACTCA
IκBα	Forward Reverse	CTTGGGTGCTGATGTCAATG ACCAGGTCAGGATTTTGCAG
NF-κBp50	Forward Reverse	TCGCTCAGCTGCACTCTATG GGGGACAGCGACACCTTTTA
NF-κBp65	Forward Reverse	CAAGTGGCCATTGTGTTCCG TGGCGATCATCTGTGTCTGG
CD16	Forward Reverse	TTTGGACACCCAGATGTTTCAG GTCTTCCTTGAGCACCTGGATC
CD32	Forward Reverse	AATCCTGCCGTTCCTACTGATC GTGTCACCGTGTCTTCCTTGAG
CD80	Forward Reverse	CCTCAAGTTTCCATGTCCAAGGC GAGGAGAGTTGTAACGGCAAGG
CD86	Forward Reverse	ACGGAGTCAATGAAGATTTCCT GATTCGGCTTCTTGTGACATAC
iNOS	Forward Reverse	AGGTTGTCTGCATGGACCAG GCTGGGACAGTCTCCATTCC
MHC II	Forward Reverse	CTGTCACGGTCGAGTGGAAA CCTGTTGGCTGAAGTCCAGA
Arg1	Forward Reverse	GCTTGCTTCGGAACTCAAC CGCATTCACAGTCACTTAGG
CD206	Forward Reverse	CCTATGAAAATTGGGCTTACGG CTGACAAATCCAGTTGTTGAGG
GAPDH	Forward Reverse	GGGTGTGAACCACGAGAAAT CCTTCCACAATGCCAAAGTT

**Table 2:** Primary antibodies used in WB.

Antibody	Source	Company	Code	Dilution
Anti-MyD88	Rabbit	FineTest (Wuhan, China)	FNab10314	1:2000
Anti-TRAF6	Rabbit	FineTest (Wuhan, China)	FNab08921	1:2000
Anti-CD80	Rabbit	Wanleibio (Shenyang, China)	WL02639	1:1000
Anti-CD86	Rabbit	HUABIO (Hangzhou, China)	ET1606-50	1:10,000
Anti-MHC II	Rabbit	Proteintech (Rosemont, IL, USA)	16109-1-AP	1:4000
Anti-Arg1	Rabbit	Wanleibio (Shenyang, China)	WL02825	1:2000
Anti-CD206	Rabbit	HUABIO (Hangzhou, China)	HA722892	1:2000
Anti-β-actin	Rabbit	Proteintech (Rosemont, IL, USA)	66009-1-Ig	1:50,000

**Table 3:** Primary antibodies used in immunofluorescence assays.

Antibody	Source	Company	Code	Dilution
Anti-MyD88	Rabbit	FineTest (Wuhan, China)	FNab10314	1:200
Anti-TRIF	Rabbit	Affinity (Cincinnati, OH, USA)	DF6289	1:200
Anti-TRAF6	Rabbit	FineTest (Wuhan, China)	FNab08921	1:200
Anti-IKKα+β	Rabbit	Beyotime (Shanghai, China)	AF2221	1:500
Anti-IκBα	Rabbit	Affinity (Cincinnati, OH, USA)	AF5002	1:200
Anti-NF-κBp65	Rabbit	Affinity (Cincinnati, OH, USA)	AF5006	1:200
Anti-CD80	Rabbit	Wanleibio (Shenyang, China)	WL02639	1:200
Anti-CD86	Rabbit	HUABIO (Hangzhou, China)	ET1606-50	1:500
Anti-iNOS	Rabbit	Proteintech (Rosemont, IL, USA)	18985-1-AP	1:500
Anti-MHC II	Rabbit	Proteintech (Rosemont, IL, USA)	16109-1-AP	1:500
Anti-Arg1	Rabbit	Wanleibio (Shenyang, China)	WL02825	1:500
Anti-CD206	Rabbit	HUABIO (Hangzhou, China)	HA722892	1:500

## Data Availability

The original contributions presented in this study are included in the article. Further inquiries can be directed to the corresponding author.
